# NLRP3-Mediated Neuroinflammation Participates in Resilience to PSD in C57BL/6 Mice

**DOI:** 10.3390/biomedicines14020297

**Published:** 2026-01-29

**Authors:** Huikang Fu, Mengqing Xiong, Qi Xu, Xiaonan Liu, Xiaohui Chen, Ying Su, Zuotian Wu

**Affiliations:** 1 Department of Neurology, Union Hospital, Tongji Medical College, Huazhong University of Science and Technology, Wuhan 430022, China; fuhuikang00521@foxmail.com (H.F.);; 2 Department of Respiratory and Critical Care Medicine, Renmin Hospital of Wuhan University, Wuhan 430060, China; meqiwh@163.com; 3 Department of Critical Care Medicine, Union Hospital, Tongji Medical College, Huazhong University of Science and Technology, Wuhan 430022, China

**Keywords:** post-stroke depression, depression resilience, NLRP3, neuroinflammation, hippocampus

## Abstract

**Background:** Post-stroke depression (PSD) is a common complication of stroke, in which neuroinflammation plays a crucial role. Depression resilience has garnered significant attention in recent years, yet its relevance to PSD remains unexplored. **Methods:** In this study, we assessed emotional behaviors in C57BL/6 mice subjected to stereotaxic injection of endothelin-1 (ET1). Subsequently, hippocampal samples were collected to analyze the levels of NLRP3/NF-κB, along with microglial activation in the hippocampus. **Results:** The results showed that ET1 injection induced depressive-like behaviors in a subset of rodents. However, a subset of mice showed no statistically significant differences from the control group in measures from the open field test (OFT), elevated plus maze (EPM), tail suspension test (TST), and forced swim test (FST), demonstrating depression resilience to PSD. Furthermore, PSD animals exhibited activated microglia and NLRP3/NF-κB activation, whereas these markers in the resilient group showed no significant difference compared to controls. **Conclusions:** This study provides evidence that depression resilience exists in a PSD model, and it suggests that the microglia–NLRP3 signaling axis may participate in this resilient phenotype. These findings offer potential intervention targets for the clinical prevention and treatment of PSD.

## 1. Introduction

Post-stroke depression (PSD) is a common and disabling complication among stroke survivors, representing a distinct subtype of depression [1,2]. Primarily characterized by persistent low mood and loss of interest, PSD is one of the most prevalent neuropsychiatric sequelae of cerebrovascular events. The latest data indicates that the cumulative incidence of PSD within five years after stroke is 59.4% [3], occurring during the acute phase (<1 month, 33%), intermediate phase (1–6 months, incidence 33%), and recovery phase (>6 months, 34%) [4]. PSD is associated with poor functional outcomes, higher long-term mortality, and increased healthcare burden [5,6].

Existing research indicates that neuroinflammation plays a pivotal role in the pathogenesis of PSD [7,8], with abnormal activation of the NLRP3 inflammasome considered a key molecular link between post-stroke brain injury and depressive symptoms [9,10]. The neuroinflammatory cascade triggered by stroke involves microglial activation, astrocytic response, dysregulation of the NF-κB pathway, and disruption of hypothalamic–pituitary–adrenal axis function. Notably, activation of the NLRP3 inflammasome-IL-1β pathway following ischemic events has been shown to promote neurodegenerative processes [11,12]. Clinical observations reveal that the vast majority of immune proteins that correlate with mood are elevated with worse mood, and of those that are reduced, several have anti-inflammatory activities, suggesting an overactivation of the immune system in chronic PSD [13]. This immune hyperactivation may contribute to depressive symptoms through mechanisms such as altered serotonin activity [14].

At the molecular mechanism level, NLRP3 inflammasome activation leads to caspase-1 cleavage [10]. Animal studies indicate increased expression of NLRP3, ASC, and cleaved caspase-1 in the ischemic region following cerebral ischemia [12]. Astrocyte-specific NLRP3 knockout experiments have confirmed that inhibiting this pathway reduces pathogenic astrocytes and significantly improves depressive-like behavior [15]. These findings collectively support the central role of the NLRP3 inflammasome in PSD. Although NLRP3 inhibitors are still in the early stages of clinical application for treating depression, modulating NLRP3 inflammasome activity in microglia is considered a promising therapeutic approach [16]. By targeting NLRP3 inflammasome inhibition, dual effects of neuroprotection (mitigating ischemia–reperfusion injury) and antidepressant action (modulating neuroinflammation) may be achieved simultaneously [17,18].

With the rise of positive psychology, depression resilience has gained significant attention both domestically and internationally in recent years. Under stress, most individuals exhibit discomfort such as depression and anxiety, but a subset resists the onset of mental disorders. This unique phenomenon is termed depression resilience [19]. Depression resilience represents a positive, self-protective adaptive response of the body, offering novel insights for the prevention and treatment of neuropsychiatric disorders [20]. Stress is a primary risk factor for depression and a common modeling strategy in depression animal models [21,22]. However, this phenomenon has not been formally reported in PSD research. Epidemiological data show that while approximately one-third of stroke patients develop PSD, two-thirds remain free of depressive symptoms, suggesting the potential existence of depression resilience in PSD.

The medial prefrontal cortex (mPFC) is a critical hub in depression-related circuitry. Inducing localized ischemic damage via ET1 microinjection in this region reliably produces depression-like behaviors, anxiety, and cognitive deficits in mice, effectively modeling post-stroke depression (PSD). This approach offers high specificity, allowing targeted study of mPFC dysfunction and its role in PSD pathogenesis and treatment [23,24,25].

Therefore, this study aimed to investigate whether depression resilience occurs in PSD and whether it involves the NLRP3 inflammasome pathway. Using a PSD mouse model, we explored the potential mechanisms underlying resilience, with a focus on NLRP3 and microglia activation. Our goal is to identify novel strategies for PSD prevention and treatment and to elucidate the therapeutic potential of targeting NLRP3.

## 2. Materials and Methods

### 2.1. Animals

Thirty male C57BL/6J mice aged 6–8 weeks were purchased from Wuhan Hualianke (Wuhan, China). Mice were housed in a Specific Pathogen Free (SPF) environment within a barrier facility. All animals were housed under standard conventional conditions (room temperature 22 ± 1 °C, humidity 55 ± 5%, ad libitum feeding) with a 12 h light/dark cycle (lights on from 6:00 AM to 6:00 PM), and they had free access to food and water. There were no differences in age, body weight, sex, or other exposure factors between groups. Mouse cages measured 320 × 215 × 170 mm and could house 6–8 mice. Animals underwent environmental adaptation for 2 weeks prior to the experiment. Food, water, and cages were sterilized before use. Animal procedures were conducted in accordance with the Regulations on the Management of Laboratory Animals issued by the State Science and Technology Commission of the People’s Republic of China.

### 2.2. Establishment of the PSD Model

Mice were anesthetized via 3% isoflurane (RWD life science, Wuhan, China) for induction of anesthesia and 1.5% isoflurane for maintenance. Bottom heating was used to stabilize the body temperature. After confirming anesthesia by placing the mouse supine and observing no response, the scull was secured in a stereotaxic frame. The scalp was incised, Bregma was located, then the X, Y, and Z axes were adjusted to zero, and the skull was leveled. Injections were administered to the medial prefrontal cortex (mPFC). The injection coordinates were as follows: AP: 1.5 mm, ML: 0.5 mm, DP: 2.6 mm; and AP: 2.0 mm, ML: 0.5 mm, DP: 2.4 mm. A hole was drilled into the skull to expose the cortex. Recent studies indicate that endothelin-1 (ET1) administered at 2 μg/μL is viable for model formation in mouse models, though its stability proves inferior to that observed in rats. This disparity may stem from the reduced number of endothelin receptors in mice. Consequently, we have opted for a higher concentration of 2.5 μg/μL compared to conventional dosages [25,26,27]. A total of 1 μL of 2.5 μg/μL endothelin-1 (ET1) (HY-P0202, MCE, dissolved in PBS) was injected across the two sites, with 0.5 μL administered at each location. Each site received a slow injection at a rate of 0.1 μL/min. The needle was retained for 5 min post-injection. Post-surgery, mice were placed in animal cages for feeding upon awakening. Behavioral validation for depressive-like behavior was conducted one week post-surgery in model mice. Mice in the PBS group received equivalent volumes of PBS.

### 2.3. Behavioral Tests

**Figure 1:**
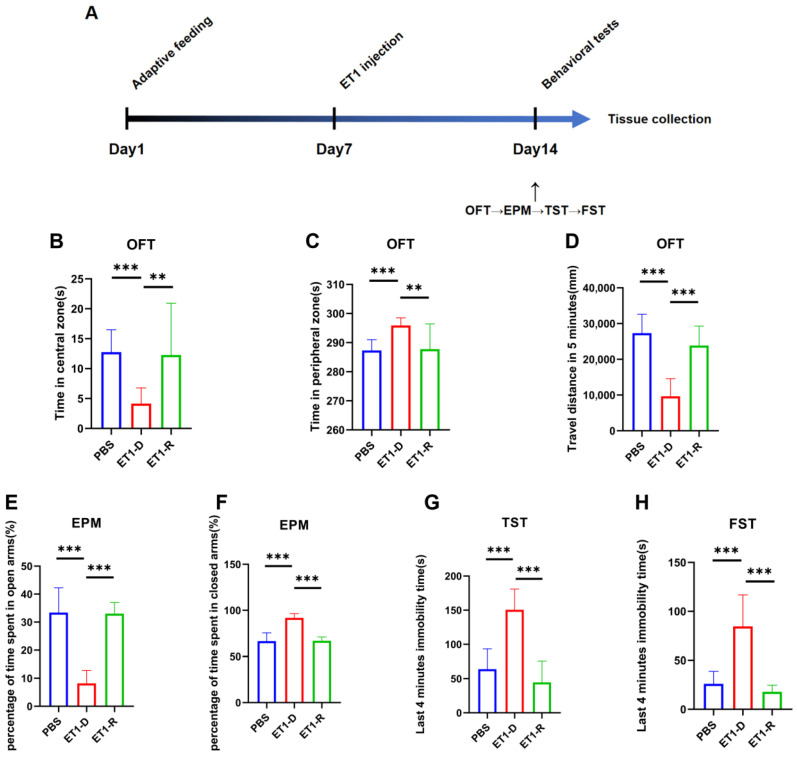
Behavioral assessment of acute PSD induced by ET1 injection. (**A**) Schematic timeline of the ET1 injection experiment. (**B**,**C**) Area preference in the open field test (OFT). (**D**) Total distance traveled by mice in the OFT within 5 min. (**E**,**F**) Area preference of mice in the elevated plus maze (EPM). (**G**) Comparison of freezing behavior duration during the final four minutes of the 6 min TST between PBS and ET1 groups. (**H**) Comparison of freezing behavior duration during the final four minutes of the 6 min FST between PBS and ET1 groups. Data are expressed as mean ± SEM. Groups included: PBS, *n* = 12; ET1-D, *n* = 12; ET1-R, *n* = 8. Compared to saline-treated animals: ** *p* < 0.01, *** *p* < 0.001.

One week after PSD surgery modeling, behavioral experiments were conducted on the model mice to determine the presence of depressive-like behaviors. Based on the experimental results, a depression resilience group and a depression group were selected. The flowchart of this experiment is shown in Figure 1A. The final groupings were as follows: PBS group, *n* = 10; ET1-depression (ET1-D) group, *n* = 12; ET1-depression-resilience (ET1-R) group, *n* = 8.

#### 2.3.1. Open Field Test

The OFT experiment was conducted in a square, solid-colored, opaque cage measuring 50 cm in length, 50 cm in width, and 35 cm in height. The software configured the center-to-periphery ratio of the open field as a square pattern with an area ratio of 0.4. Each mouse was placed in the center of the open field with its back toward the experimenter, and the camera began recording simultaneously. The software automatically tracked parameters including total distance traveled, distance traveled within the central zone, duration spent in the central zone, and immobility time over a 5 min period via the overhead camera. Equipment was cleaned with 75% ethanol after each trial. Anilab software (Ver 4.1, Anilab Scientific Instruments Ltd. Co, Ningbo, China) was used to analyze the traveling distance and average speed of the mice.

#### 2.3.2. Elevated Plus Maze Test

The EPM platform consists of two open arms (35 cm × 5 cm) perpendicular to two closed arms of identical dimensions (35 cm × 5 cm), forming a cross shape with a small central square (5 cm × 5 cm) between the arms. The maze stands 50 cm above the ground. Each animal is placed in the center of the maze with its back toward the experimenter and facing the open arms oriented in the same direction. A video tracking system records data including time spent, distance traveled, and rest periods within the open arms, closed arms, and center area over a 5 min period. The apparatus is cleaned with 75% ethanol after each trial. Anilab software (Anilab Scientific Instruments Ltd. Co, Ningbo, China) was used to analyze the traveling distance and average speed of the mice.

#### 2.3.3. Tail Suspension Test

In the TST experiment conducted in a darkened room, the tip of each mouse’s tail was secured with adhesive tape (a plastic tube approximately 3 cm in diameter could be fitted over the tail to prevent the mouse from curling its body and climbing). The mouse was suspended 40 cm above the ground. A camera recorded all behaviors of each mouse over a 6 min period, with the first 2 min designated as an acclimation phase. For the last four minutes, the total duration of immobility (complete lack of movement other than respiration) was assessed by a trained observer blinded to the groupings of the animals.

#### 2.3.4. Forced Swimming Test

The FST experiment requires placing mice with their backs facing the experimenter in a transparent cylindrical water tank measuring 50 cm in height and 30 cm in diameter. The water depth must exceed the maximum distance from the mouse’s head to its tail, ensuring the mouse cannot touch the bottom. A camera recorded all behaviors of each mouse over a 6-min period, with the first 2 min designated as an adaptation phase. For the last four minutes, the total duration of immobility (complete lack of movement other than respiration) was assessed by a trained observer blinded to the groupings of the animals.

### 2.4. Tissues Collection and Analysis

After behavioral testing, all mice were euthanized. Based on behavioral data analysis results, they were grouped, with 4 mice randomly sampled from each group for hippocampal extraction for Western blot analysis. The remaining mice underwent whole-brain perfusion for fluorescence staining.

#### 2.4.1. Protein Tissue Collection

Euthanasia of mice via cervical dislocation was performed by trained personnel in a quiet environment, avoiding the presence of other mice. The procedure was as follows: Using the thumb and index finger of the dominant hand, firmly grasp the base of the mouse’s tail, allowing its forelimbs to cling to a support surface while the body remains naturally extended. With the knuckle of the index or middle finger of the non-dominant hand, firmly press against the back of the mouse’s head and neck as a fulcrum. The hand holding the tail must then decisively, swiftly, and forcefully pull the mouse upward and backward. Immediate confirmation of death must be performed after the procedure. Incise the skull along the midline using surgical scissors, working gently to avoid damaging brain tissue. Use hemostatic forceps to cut the skull from the inside out, then slowly remove the skull from bottom to top to expose the entire brain. Use eye forceps to remove the meninges and blood vessels from the brain surface. Remove the brain tissue from the skull base, immerse it in 0–4 °C saline for 30 s, and place it on an ice pack to isolate the mouse hippocampal tissue. Immediately transfer the tissue to a −80 °C freezer for storage.

#### 2.4.2. Western Blot

Equal amounts of protein samples were separated on a 12% SDS-PAGE gel and transferred to a polyvinylidene fluoride membrane (Merck Millipore, Billerica, MA, USA). The following primary antibodies were used: NLRP3, 1:1000, NLRP3 (D4D8T) Rabbit mAb #15101, CST; Caspase-1, 1:1000, A0964, AbClonal; p65, 1:1000, 8242S, CST; Actin, 1:10,000, 66009-1-ig, Proteintech; IBA1, 1:1000, 019-19741, wako. The membrane was incubated overnight at 4 °C with the primary antibody. After washing away the primary antibody the following day, the membrane was incubated with the secondary antibody. β-actin served as the internal control for total protein. Band intensities were measured using ImageJ software (Ver1.46r, NIH, Bethesda, MD, USA).

#### 2.4.3. Transcardial Perfusion

The following procedure was used for transcardial perfusion: First, place the mouse in the induction chamber and administer deep anesthesia with 3% isoflurane (R510-22-10, RWD). Once the righting reflex has completely disappeared, transfer the mouse to the operating table and maintain anesthesia with 1.5% isoflurane via mask inhalation. Subsequently, secure the mouse in the supine position, rapidly incise the thoracic cavity to expose the heart, insert the perfusion needle through the left ventricle into the ascending aorta, and secure it while simultaneously incising the right atrial appendage to create an outflow tract. Rapidly perfuse with pre-chilled saline until the liver turns white and fluid exiting the right atrium becomes clear. Switch to 4% paraformaldehyde fixative for perfusion; at this point, the animal’s body rapidly stiffens. After perfusion completion, harvest the required tissues and proceed with post-fixation. The brains were post-fixed in 4% paraformaldehyde overnight. After sectioning using an ice microtome, suitable brain slices containing the hippocampus were selected.

#### 2.4.4. Immunofluorescence

Following membrane disruption and blocking with 3% BSA mixed with 0.5% triton in PBS for 1 h, the slices were sequentially incubated with primary antibodies at 4 °C overnight. After washing away the primary antibody the following day, the slices were incubated with the secondary antibody. The following primary antibodies were used: Iba1 (1:500, 019-19741, Wako), CD68 (1:500, ab53444, Abcam), CD11c (1:500, 97585, CST). After imaging with a confocal microscope, average fluorescence intensity was measured using ImageJ software.

### 2.5. Statistical Analyses

Data were expressed as mean ± SEM. Graph-Pad Prism 8.0 software for Windows (GraphPad Software, Inc, San Diego, CA, USA) was used for statistical analysis and graphing. Data were analyzed via one-way ANOVA, followed by the Tukey’s for post hoc multiple range comparisons. *p* < 0.05 was considered statistically significance. Variables with *p*-values < 0.05 were considered statistically significant.

## 3. Results

### 3.1. ET1-Induced PSD, but with Depression Resilience Observed

We injected the vasoconstrictor ET1 into the mPFC of mice. On day 7 post-ET1 injection, we conducted behavioral tests to assess depression- and anxiety-like behaviors, sequentially performing the OFT, EPM, TST, and FST (Figure 1A).

This study found that among animals receiving ET1 injections into the mPFC, some did not exhibit depressive-like behaviors. Specifically, for all ET1 injected mice, we calculated the sum of the last 4 min of immobility times from both the TST and FST. Since the maximum immobility time observed in the PBS control group for either test was approximately 100 s, we established a clear threshold: ET1-injected mice with a combined TST and FST immobility time of less than 100 s were classified into the resilient (ET1-R) group. Mice exceeding this threshold were classified as depressed (ET1-D). This criterion was applied first. In previous studies on chronic stress-induced depression, mice subjected to chronic stress modeling were categorized into the CRS-low group based on immobility time in the forced swim test being less than 50 s [28]. The final groupings were as follows: PBS group, *n* = 10; ET1-D group, *n* = 12; ET1-R group, *n* = 8.

Following this classification based on TST/FST, we then statistically compared the resulting groups, PBS, ET1-D, and ET1-R on the other behavioral tests. As shown in the results, the ET1-R group showed no significant difference from the PBS group in the OFT (center/periphery preference and total distance) or in the EPM (percentage of time in open/closed arms). In contrast, the ET1-D group displayed significant anxiety-like and depressive-like profiles in these tests compared to both the PBS and ET1-R groups.

### 3.2. Microglia Were Activated in PSD but Reduced in Resilience to PSD Mice

**Figure 2:**
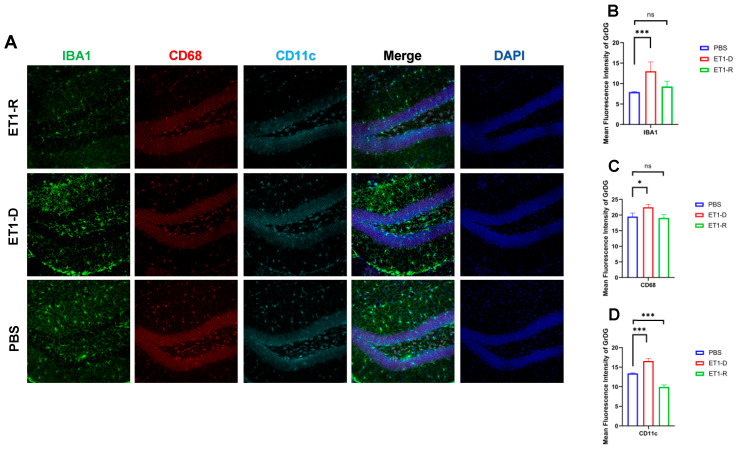
Expression of microglia in the hippocampal GrDG region. (**A**) IBA1, CD68, and CD11c immunofluorescence staining results in the PBS group, ET1-D group, and ET1-R group. NLRP3 immunofluorescence staining results in the GrDG region. (**B**) Analysis of average fluorescence intensity of IBA1 in the GrDG region. (**C**) Analysis of average fluorescence intensity of CD68 in the GrDG region. (**D**) Analysis of average fluorescence intensity of CD11c in the GrDG region. Data are expressed as mean ± SEM. Groups included: PBS, *n* = 6; ET1-D, *n* = 8; ET1-R, *n* = 4. Compared to PSD animals: ns *p* > 0.05, * *p* < 0.05, *** *p* < 0.001.

To investigate the potential mechanisms underlying PSD resilience, we further examined the expression of hippocampal microglia. As shown in Figure 2, our study revealed significantly increased expression of IBA1 and CD11c in the Granule cell layer of the Dentate Gyrus (GrDG) region of ET1-induced depression-like animals (*p* < 0.001, Figure 2). However, compared with the PBS group, no statistically significant differences were observed in these indicators between the ET1-R group and the PBS group (*p* > 0.05).

**Figure 3:**
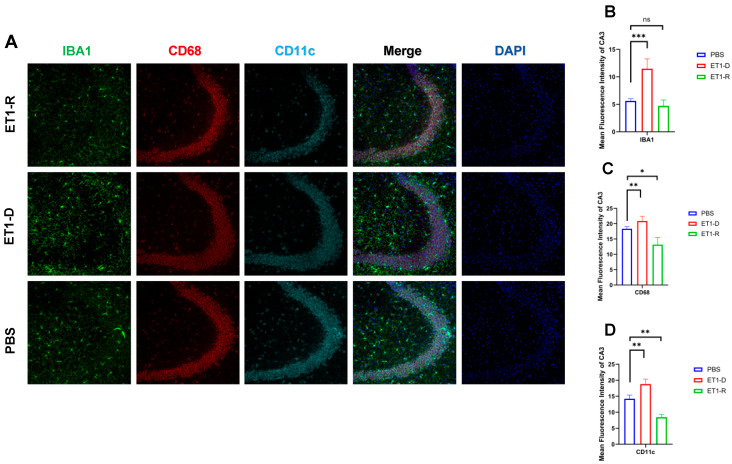
Expression of microglia in the hippocampal CA3 region. (**A**) Immunofluorescence staining results for IBA1, CD68, CD11c, and NLRP3 in the CA3 region among the PBS group, ET1-D, and ET1-R group. (**B**) Analysis of average fluorescence intensity of IBA1 in the CA3 region. (**C**) Analysis of average fluorescence intensity of CD68 in the CA3 region. (**D**) Analysis of average fluorescence intensity of CD11c in the CA3 region. Data are expressed as mean ± SEM. Groups included: PBS, *n* = 6; ET1-D, *n* = 8; ET1-R, *n* = 4. Compared to PSD animals: ns *p* > 0.05, * *p* < 0.05, ** *p* < 0.01, *** *p* < 0.001.

As shown in Figure 3, in the hippocampal CA3 region, ET1 injection induced elevated IBA1 expression compared to the PBS group (*p* < 0.01, Figure 3B), without affecting CD68 and CD11c expression (*p* > 0.05, Figure 3C,D). However, IBA1 expression in the hippocampal CA3 region of the ET1-R group did not differ from that of the PBS group (*p* > 0.05). Furthermore, CD68 and CD11c expression in the ET1-R group was significantly lower than that in the PBS group (all *p* < 0.05).

**Figure 4:**
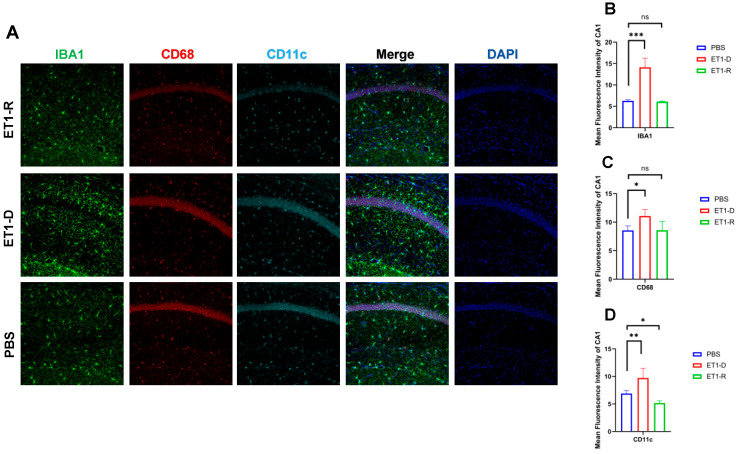
Expression of microglia in the hippocampal CA1 region. (**A**) IBA1, CD68, and CD11c immunofluorescence staining results in the PBS group, ET1-D group, and ET1-R group. NLRP3 in the CA1 region. (**B**) Analysis of average fluorescence intensity of IBA1 in the CA1 region. (**C**) Analysis of average fluorescence intensity of CD68 in the CA1 region. (**D**) Analysis of average fluorescence intensity of CD11c in the CA1 region. Data are expressed as mean ± SEM. Groups included: PBS, *n* = 6; ET1-D, *n* = 8; ET1-R, *n* = 4. Compared to PSD animals: ns *p* > 0.05, * *p* < 0.05, ** *p* < 0.01, *** *p* < 0.001.

As shown in Figure 4, in the hippocampal CA1 region, ET1 injection induced elevated IBA1 expression (*p* < 0.01, Figure 4B) compared to the PBS group, without affecting CD68 and CD11c expression (*p* > 0.05, Figure 4C,D). However, in the ET1-R group, IBA1 and CD68 expression in the hippocampal CA1 region did not differ from the PBS group (*p* > 0.05). Furthermore, CD11c expression was significantly lower than in the PBS group (all *p* < 0.05).

**Figure 5:**
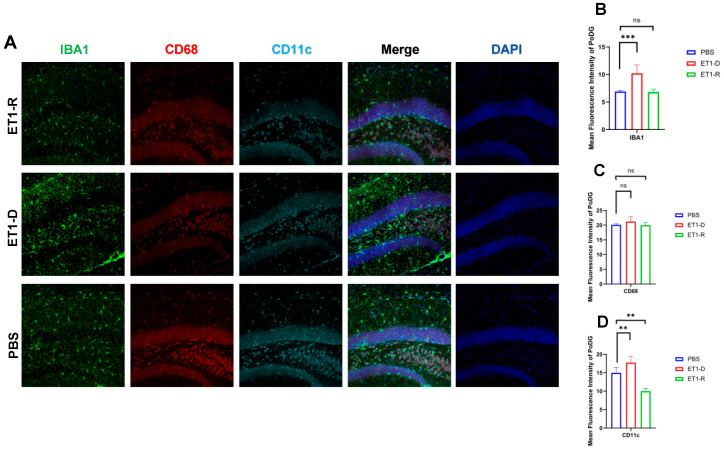
Expression of microglia in the PoDG region of the hippocampus. (**A**) Immunofluorescence staining results for IBA1, CD68, CD11c, and NLRP3 in the PoDG region among the PBS group, ET1-depressed group, and ET1–depression–resilience group. (**B**) Analysis of average fluorescence intensity of IBA1 in the PoDG region. (**C**) Analysis of average fluorescence intensity of CD68 in the PoDG region. (**D**) Analysis of average fluorescence intensity of CD11c in the PoDG region. Data are expressed as mean ± SEM. Groups included: PBS, *n* = 6; ET1-D, *n* = 8; ET1-R, *n* = 4. Compared to PSD animals: ns *p* > 0.05, ** *p* < 0.01, *** *p* < 0.001.

As shown in Figure 5, in the Polymorphic layer of the Dentate Gyrus (PoDG) region, ET1 injection induced elevated IBA1 expression (*p* < 0.01, Figure 5B) compared to the PBS group, without affecting CD68 and CD11c expression (*p* > 0.05, Figure 5C,D). However, in the ET1-R group, IBA1 and CD68 expression in the hippocampal CA3 region showed no difference from the PBS group (*p* > 0.05). Furthermore, CD11c expression was significantly lower than in the PBS group (all *p* < 0.05).

CD68 and CD11c were not significantly increased in the ET1-D group. We suggest this may reflect the specific temporal dynamics or regional characteristics of microglial phenotype expression following ET1-induced ischemia, and that IBA1 (a general marker) may be more sensitive to the initial activation state in this model. In Figure 2, Figure 3, Figure 4 and Figure 5, the nuclear staining signal of CD68 and CD11c was partially excluded in the quantifications of fluorescence intensity.

### 3.3. NLRP3/NF-κB Activation Occurred in ET1-Induced PSD

**Figure 6:**
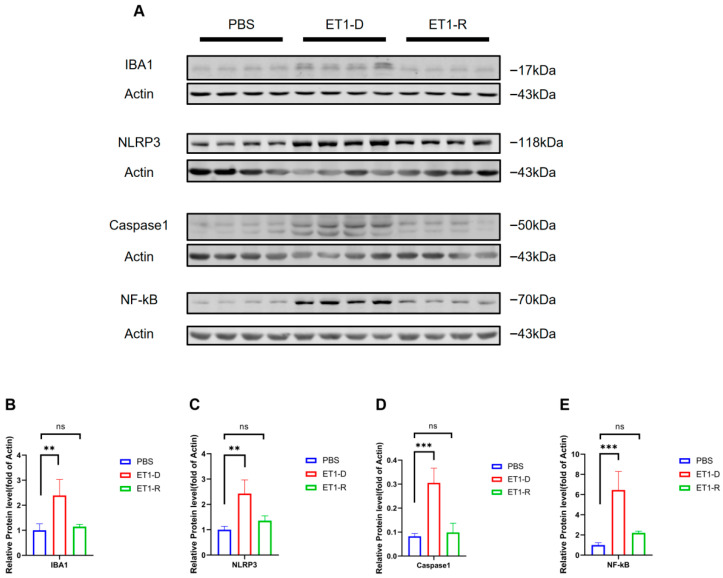
NLRP3/NF-κB activation occurs in ET1-induced PSD but is suppressed in the resilience group. (**A**) Western blot analysis of IBA1, NLRP3, Caspase1, and NF-κB expression in PBS, ET1-depressed, and ET1-resilience groups. (**B**) Gray-scale analysis of IBA1. (**C**) Gray-scale analysis of NLRP3. (**D**) Gray-scale analysis of Caspase1. (**E**) Gray-scale analysis of NF-κB. Each lane represents a single mouse sample. Data are expressed as mean ± SEM. Groups included: PBS, *n* = 4; ET1-D, *n* = 4; ET1-R, *n* = 4. Compared to PSD animals: ns *p* > 0.05, ** *p* < 0.01, *** *p* < 0.001.

We further examined the protein levels of Iba1 and NLRP3/NF-κB, markers of microglial activation, in hippocampal tissue. As shown in Figure 6, compared with the PBS group, the Iba1 protein level was significantly elevated in the ET1-D group (*p* < 0.05, Figure 6B), indicating that stereotaxic injection of ET1 activates microglial expression. Furthermore, compared with the PBS group, NLRP3, caspase-1, and NF-κB expression were significantly upregulated in the ET1-D group (*p* < 0.05, Figure 6C–E). However, no statistically significant differences were observed in these markers between the ET1-R group and the PBS group (all *p* > 0.05, Figure 6B–E).

## 4. Discussion

This study investigated whether depression resilience exists in PSD and explored its association with neuroinflammation. We found that stereotactic injection of ET1 into the brain induced depressive-like behaviors in a subset of mice [25,26,27]. However, another subset of mice exhibited behavioral profiles indistinguishable from PBS group in measures from the OFT, EPM, TST, and FST, indicating the occurrence of depression resilience. Furthermore, the ET1-D group exhibited activated microglia and NLRP3/NF-κB activation. However, the ET1-R group showed no differences in these markers compared to PBS group, suggesting that depression resilience in PSD may be closely associated with reduced microglial reactivity and inhibition of the NLRP3 inflammasome signaling pathway. This finding indicates that depression resilience may exert a protective effect by modulating neuroinflammatory responses.

PSD is one of the most common neuropsychiatric complications following stroke [29]. Epidemiological studies indicate its prevalence can reach 30–50%, significantly impacting patients’ neurological recovery and quality of life [30,31]. Previous clinical research suggests not all stroke patients develop depression, with marked heterogeneity observed between individuals; some patients exhibit innate resilience to depression [1,2,3,29,30]. Therefore, we propose the hypothesis that endogenous depression resilience mechanisms exist after stroke, potentially related to the regulation of neuroinflammation. Resilience to neuroinflammation may represent a key mechanism enabling individuals to effectively avoid the development of PSD [32].

Our study confirmed the existence of PSD resilience using an ET1-induced acute ischemic stroke model [25,26,27]. First, following ET1 injection, some mice exhibited pronounced depressive-like behaviors compared to the PBS group. These included a preference for the peripheral area in the OFT, a preference for the closed arms in the EPM, and prolonged immobility times in both the TST and FST, consistent with the PSD behavioral phenotype. Conversely, a subset of mice exhibited no significant depressive-like behaviors despite identical stroke exposure, showing no statistically significant differences in behavioral metrics compared to the PBS group. These mice were defined as PSD-resilient. This finding indicates substantial individual variation in behavioral responses under comparable pathological conditions, establishing an animal model foundation for further mechanistic investigations.

Microglia are central mediators of post-stroke neuroinflammation and have been strongly linked to PSD pathogenesis [6,8,33,34]. Our findings reveal that in stroke-resilient mice, microglia reactivity is significantly reduced compared to stroke-depressed mice, accompanied by decreased expression of NLRP3, caspase-1, and NF-κB. As a crucial molecular platform in innate immunity, NLRP3 inflammasome activation triggers caspase-1 cleavage and the maturation/release of proinflammatory cytokines IL-1β and IL-18, exacerbating neuroinflammation and promoting depressive-like behaviors [10,35,36]. Therefore, our data suggest that resilience may be linked to an inherent capacity to limit this specific neuroinflammatory cascade.

An interesting observation warrants further investigation. As astutely noted, protein levels of NLRP3 and NF-κB in the ET1-R group, while not statistically elevated, appeared marginally higher than in PBS controls in the original Western blot (Figure 6). This observation, coupled with cleaved caspase-1 levels indistinguishable from controls, may suggest a distinct neuroinflammatory state in resilient individuals. Rather than full inflammasome activation (as seen in the ET1-D group), this could reflect a primed or intermediate state where the inflammatory machinery is present but its downstream execution (caspase-1 cleavage) is effectively restrained. This aligns with the concept of resilience not as a mere absence of challenge but as an active adaptive process involving regulated signaling. Future studies should investigate the precise regulatory checkpoints that prevent this primed state from progressing to full NLRP3 inflammasome assembly and activation in resilient subjects.

The functional interplay between microglia and the NLRP3 inflammasome is well-documented, often forming a feed-forward loop that amplifies inflammation [37,38,39]. The observed suppression of this loop in resilient mice suggests that upstream regulatory mechanisms may exist to modulate microglial activation state and subsequent NLRP3 activity. Identifying these upstream regulators could reveal novel targets for promoting resilience [40,41,42].

This study has several important limitations. First, we used only male C57BL/6 mice, and the influence of sex or genetic strain on resilience remains unexplored. Most critically, the mechanistic insights regarding NLRP3 remain correlative. While our data highlight an association between the resilient phenotype and a dampened NLRP3 response, they do not establish causality. Direct validation of the pathogenic role of NLRP3 in our PSD model, through the use of *NLRP3* gene knockout mice or specific pharmacological inhibitors (e.g., MCC950), is necessary to confirm whether inhibiting this pathway is sufficient to confer a resilient phenotype. Future studies employing these tools are essential to move from correlation to causation and to solidify NLRP3 as a beneficial therapeutic target. Investigating synaptic markers will be important in our future work to link inflammatory changes to functional neural outcomes.

Additionally, our study did not measure downstream cytokines of the NLRP3 inflammasome pathway, such as pro-IL-1β and mature IL-1β (or IL-18). Although we observed alterations in NLRP3 and caspase-1 expression, direct assessment of these effector cytokines would provide a more complete understanding of inflammasome activity and its functional output in PSD resilience. Future studies incorporating cytokine profiling are warranted to fully delineate the NLRP3–IL-1β axis in this context.

Finally, translating these findings to human PSD requires validation in clinical cohorts.

## 5. Conclusions

In conclusion, this study provides evidence for the existence of depression resilience in an ET-1-induced mouse model of PSD. Resilient mice displayed normal affective behavior alongside reduced hippocampal microglial activation and attenuated NLRP3/NF-κB pathway activity. These findings suggest that endogenous resilience mechanisms may mitigate PSD development by limiting neuroinflammation, with the microglia–NLRP3 axis representing a potential mechanistic correlate. This work lays a foundation for future research aimed at understanding the biological basis of resilience and developing interventions that target neuroinflammatory pathways to prevent or treat PSD.

## Data Availability

The data presented in this study are available upon request from the corresponding author due to privacy and ethical reasons.
